# Molecular Frontiers in Transplantation-Induced Ischemia–Reperfusion Injury

**DOI:** 10.3390/ijms24043450

**Published:** 2023-02-09

**Authors:** Raphael Thuillier

**Affiliations:** 1 Biochemistry Department, CHU Poitiers, 86021 Poitiers, France; raphael.thuillier@univ-poitiers.fr; 2 Faculty of Medicine and Pharmacy, University of Poitiers, 86073 Poitiers, France; 3 INSERM U1313, IRMETIST, 86021 Poitiers, France

This Special Issue aims to summarize the most up-to-date research on ischemia–reperfusion and organ transplantation. Indeed, while organ transplantation is the best therapy to treat organ failure, its success is impeded by the unavoidable step of organ preservation which is intimately linked to ischemia–reperfusion injury (IRI). We are thus in need of novel insights into the molecular mechanisms of IRI, a necessary basis on which to develop novel approaches to better manage the transplantation process and improve outcomes [1].

In this Special Issue, four papers focus on the mechanisms of IRI:

- Exploring lung transplantation and related kidney injuries, Greite et al. [2] show that lung transplant patients suffering from post-reperfusion AKI have increased levels of cell-free hemoglobin and decreased haptoglobin, pointing towards a possible use for these biomarkers as predictors of kidney injury. Moreover, the authors further demonstrate the role of cell-free hemoglobin as a pro-injury agent in the kidneys in a mouse model, thus opening up the possibility of cell-free hemoglobin as a therapeutic target.
- The same team explored the differential impact of kidney ischemia–reperfusion and veinous/veinous extracorporeal membrane oxygenation (ECMO) [3], demonstrating that the latter was associated with a milder level of kidney injury, characterized by different kinetics of pro-inflammatory cytokine release as well as the absence of tubular function impairment and immune cell infiltration compared to IRI. Interestingly, their work revealed that ECMO induced higher expression of heme oxygenase 1 in tubular cells, suggesting higher activity of cell-free hemoglobin catabolism, thus protecting the kidney. This is not unlike previously published data on the protective role of ECMO in the context of normothermic regional perfusion [4] which is now used in France for the management of deceased after circulatory arrest donors.
- An often overlooked protagonist in IRI is hypothermia [5], which offers protection against deleterious pathways, but very low temperatures such as 4–8 °C deeply affect protein structure, lipid membrane integrity, and cytoskeletal organization [6], which are detrimental to cell survival, prompting research into mild hypothermia [7]. Regarding this issue, Kattri-Liis et al. [8] show that mild hypothermia provides bimodal protection, combining metabolism inhibition with the induction of stress tolerance pathways. The authors demonstrate that 32 °C appears to be the best preservation temperature to balance the inhibition with the activation components of hypothermia and to protect against reducing stress. Interestingly, at temperatures below that point, the HIF pathway cannot properly function, impeding a major survival pathway in the context of organ preservation.
- Another well described mechanism during IR is investigated in this Special Issue, namely oxidative stress [9]. Indeed, Spraalman et al. [10] quantify oxidative-stress-marker-free sulfhydryl groups in living donor kidney transplantation, demonstrating the involvement of oxidative stress even in what are considered to be the most favorable conditions in organ transplantation. This work further highlights the importance of oxidative stress as a viable target for therapeutics, free sulfhydryl groups measurement as a possible marker, and hence of the level of kidney injury suffered during ischemia.

The lack of proper biomarkers is indeed a difficulty in transplantation, impeding the evaluation of organ quality and thus the possibility to anticipate outcomes. This specific problem is reviewed by Lepoittevin et al. in the present Special Issue [11], with the researchers focusing particularly on high-throughput methods (i.e., the ‘omics’) and offering an overview of their pros and cons, as well as highlighting the recent development of metabolomics and the necessary care required in using these methods to obtain reliable and exploitable data for the discovery of novel biomarkers.

While biomarkers are indeed a key issue in transplantation, innovative approaches to organ care are also of paramount importance. In this Special Issue, two such innovations are presented:

- Kröger et al. [12] present a concept of liver preservation adapted for highly damaged organs, such as those from deceased after circulatory death donors. They combined venous systemic oxygen persufflation, a concept well explored for kidney [13] and liver [14] preservation, with the additive benefits of fibrinolysis through the use of streptokinase [15] in a rat model of liver transplantation. Their results show that, indeed, the two therapeutic strategies provide additive benefits to liver quality.
- Finally, Ernst et al. [16] directed their efforts not towards preservation but instead towards alleviating the intermediate period between the end of preservation and anastomosis. Indeed, while the donor organ arteries and veins are anastomosed to the recipient circulation, the organ is subjected to the stress of warm ischemia, known to enhance IRI. Through the use of thermal insulation, the authors demonstrate the benefits of controlling the temperature change during the anastomosis step of transplantation. While this proof of concept shows limited benefits due to the low injury conditions of the model, the potential for a higher level of protection for more fragile organs needs to be seriously considered, especially in the current context of organ shortages and increased reliance on marginal donors.

All in all, this Special Issue presents two very interesting reviews to provide an update on organ preservation and recent advances in omics research. The research papers herein highlight the ever-important role of temperature and oxidative stress along the donor–organ–recipient itinerary as well as point towards current issues such as oxygenation (either during preservation or at the deceased after circulatory arrest donor level).
